# Lung cancer embolization causing acute limb ischemia: a case report

**DOI:** 10.1186/s13256-023-03769-5

**Published:** 2023-02-17

**Authors:** Syed Mohammad Asim Hussain

**Affiliations:** Frimley Park Hospital, Portsmouth Road, Camberley, GU16 7UJ Frimley UK

**Keywords:** Tumor embolism, Lung cancer, Limb ischemia, Embolization, Embolectomy, Fasciotomy, Vascular

## Abstract

**Background:**

Acute tumour embolism to the popliteal artery resulting in limb-threatening ischemia is a rare complication of neoplastic disease. Generally, tumors embolize to the pulmonary circulation via the venous system. In this case, the originating tumor was a lung cancer of a large size and advanced stage that had invaded the left atrium of the heart and disseminated in the systemic circulation. The tumor likely fragmented, resulting in showering to the right popliteal artery, superior mesenteric artery, and left renal artery, which is a unique presentation of tumor embolism.

**Case report:**

We present a case of a 62-year-old Caucasian gentleman with a large left lower lobe squamous cell lung cancer that had invaded into the left atrium via the pulmonary veins. He presented with acute limb threatening ischemia. A computed tomographic angiogram revealed an occlusion of the left popliteal artery as well as embolization to the superior mesenteric artery and the right renal artery. He was started on intravenous heparin and underwent an emergency popliteal embolectomy and calf fasciotomies, which was limb saving. His fasciotomy wounds were closed after 1 week and he was discharged on anticoagulation.

**Conclusion:**

This is a rare case of tumor embolism resulting in both an embolectomy and calf fasciotomies. In the light of such cases, we suggest that tumors invading the bloodstream should be considered high risk for embolization and hypothesize that prophylactic antithrombotic therapy may avoid major morbidity.

## Introduction

Acute limb ischemia secondary to tumor embolism is a rare complication of primary neoplasm. An analysis of 877 cases of arterial embolization showed that only three cases (0.34%) were caused by tumor embolism [1]. The majority of these are caused by primary lung cancer (44.2%) or lung metastases (31.7%) [2]. Another common cause is cardiac myxoma, a benign tumor commonly originating in the left atrium. In addition to these, a wide variety of tumors have been implicated in tumor embolization including breast cancer, gastrointestinal malignancies (esophageal, liver, gastric, colorectal), urological malignancies (renal, ureteric, prostate, bladder), hematological malignancies (leukemias, lymphomas), and sarcomas.

The showering of tumor emboli has been known to cause acute occlusions at various sites including the mesenteric, brachial, common femoral, and popliteal arteries [3]. These are all vascular emergencies requiring immediate embolectomy to restore perfusion.

These patients invariably have a poor prognosis as tumor embolization indicates advanced cancer, which is the primary cause of mortality, even if the acute treatment of embolization is successful [4].

We present a case of a large primary lung cancer, which had invaded into the left atrium. This resulted in tumor embolization at multiple arterial sites and acute limb-threatening occlusion of the left popliteal artery requiring embolectomy and fasciotomy. The popliteal artery generally has a diameter of 7–8 mm in men. The completeness and severity of popliteal arterial occlusion indicates that large amounts of tumor fragments were released into the bloodstream with concomitant secondary thrombosis. This is a unique presentation of tumor embolism, in which surgical intervention and fasciotomy was required to prevent limb loss.

## Case presentation

A 62-year-old Caucasian gentleman presented to respiratory clinic with persistent cough, hemoptysis, and recurrent chest infection. He was unwell for 6 months with increased breathlessness, mild hoarseness of voice, fatigue, reduced appetite, and weight loss. A chest X-ray showed left-sided opacification and a small effusion.

**Fig. 1:**
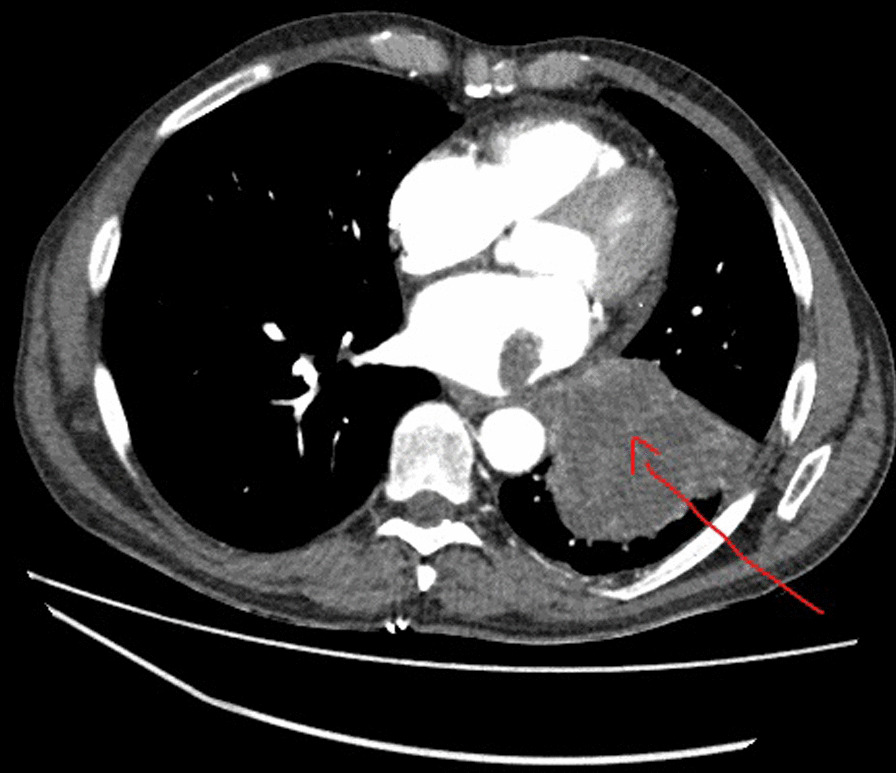
CT thorax showing left lower lobe lung cancer invading into inferior pulmonary vein and left atrium indicated by the red arrow

Computed tomography (CT) of the thorax showed a large 7 × 9 cm complex centrally necrotic lesion of the left lower lobe with distal collapse and consolidation (Fig. 1). This was associated with multiple, enlarged bilateral hilar nodes, supraclavicular node, and a thrombus in the left inferior pulmonary vein. The radiological stage was T4N3M1a/c.

He had a background of osteoarthritis, hyperlipidemia, and benign prostatic hyperplasia. He was an ex-smoker with 25–30 cigarettes/day until recently.

After 6 months, he presented to vascular surgery with sudden onset left leg pain. On examination, the left leg was cold, mottled, pale, and lacking sensation but he was able to move his toes. He had a palpable femoral pulse but an absent left popliteal pulse. He was diagnosed with acute limb ischemia and was started on intravenous heparin.

**Fig. 2:**
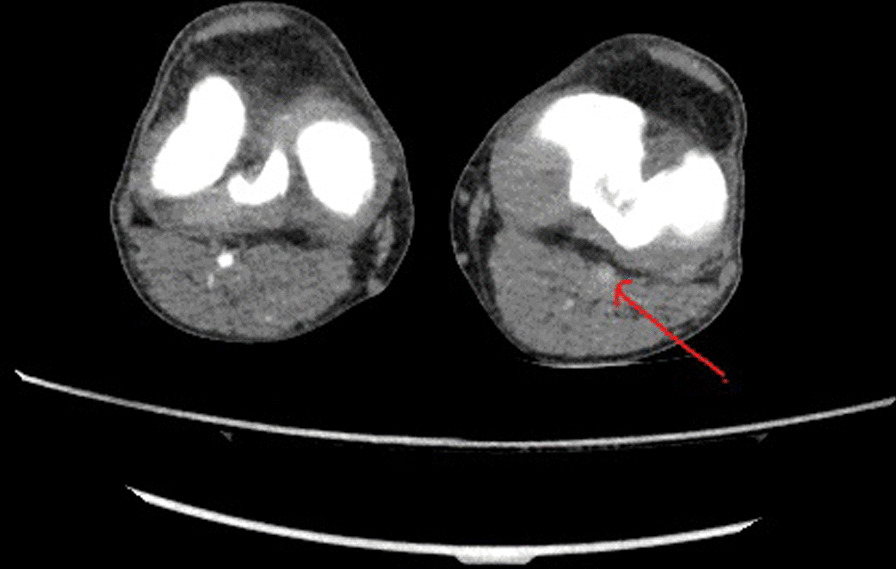
Left popliteal artery occlusion due to tumor embolism indicated by the red arrow (axial view)

The CT angiogram showed an occlusive thrombus in the mid superior mesenteric artery (SMA). The inferior mesenteric artery was patent and iliofemoral arteries were patent with no opacification of the arteries from mid-thigh onward. He was recalled for delayed phase imaging, which showed a large popliteal artery occlusion at the bifurcation with no run-off distally (Fig. 2).

**Fig. 3:**
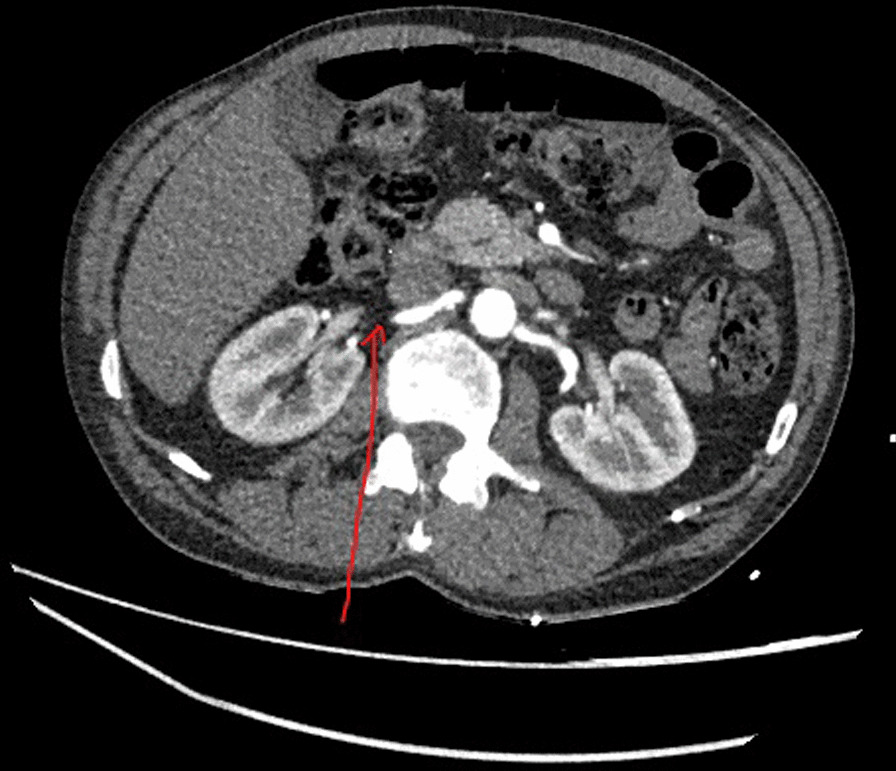
Occlusion of the right renal artery (see red arrow) due to tumor embolism leading to renal infarct

In addition, the large necrotic tumor was noted and was causing complete collapse of the left lower lobe. The tumor extended along the inferior pulmonary vein into the left atrium and appearance had worsened since previous imaging. The lower pole of the right kidney was infarcted (Fig. 3). A diagnosis of tumor embolism into the SMA, right renal artery, and left popliteal artery was made. He did not have any symptoms of abdominal pain or any signs of intestinal necrosis on the CT scan.

He underwent a left popliteal embolectomy along with calf fasciotomies. This involved open surgical exposure of the popliteal artery using a medial approach below the knee. The popliteal artery was carefully dissected free. Proximal and distal vessel control was obtained and a transverse arteriotomy was made. A Fogarty balloon catheter was used to perform the embolectomy and adequate blood flow was seen. Polypropylene suture was used to close the arteriotomy in an interrupted manner. Four-compartment fasciotomies were then performed.

Postoperative arterial duplex did not identify flow in the tibio–peroneal trunk, the posterior tibial artery, and the distal peroneal artery. There was monophasic flow in the anterior tibial artery. However, the foot was warm and well perfused. Postoperatively, he was anticoagulated with subcutaneous therapeutic dalteparin injections, 12,500 units/day. The fasciotomy wounds were closed a week later, and the patient was discharged.

The embolectomy specimen sent to the lab contained three pieces of arterial tissue and multiple fragments of gray tissue, which measured 15 × 25 × 8 mm in aggregate. Microscopic examination showed that all pieces of tissue had histological features of a recent thrombus. The pieces of gray tissue macroscopically resembled lung tissue. Immunohistochemical staining was not done to formally identify tumor cells in the specimen.

He subsequently underwent a bronchoscopy and biopsy, which confirmed squamous cell lung cancer, characterized by sheets of large, polygonal, malignant cells containing keratin. This was treated palliatively with chemotherapy and radiotherapy.

## Discussion

This is a unique case of a lung cancer embolizing to the systemic circulation via the arterial system. Generally, the literature describes tumor embolization to the pulmonary circulation via the venous system, often with an underlying gastrointestinal, breast, soft-tissue, or urological malignancy. For example, Aiyappan *et al*. described a case of pulmonary tumor embolism due to a gastric carcinoma and Murugan *et al*. reported a case of invasive ductal breast cancer with embolization to the pulmonary artery [5, 6]. Similarly, Grass *et al*. described a case of pulmonary tumor embolism due to a myxoid sarcoma originating from the thyroid gland [7]. A further example was provided by Salam *et al*. who reported a case of urothelial carcinoma with neoplastic embolization to the pulmonary artery resulting in pulmonary hypertension due to pulmonary thrombotic microangiopathy [8].

Lung cancer embolization into the systemic circulation has also been reported in the literature. In 2018, Cheema *et al*. reported a case of non-small cell lung cancer with cardiac metastases that embolized to the infra-renal aorta and bilateral common iliac arteries resulting in bilateral limb ischemia [9]. Surgical thrombectomy was performed but the patient did not survive. Togo *et al*. in 2018 reported a case of squamous cell lung cancer with left atrial invasion that embolized to the right superficial femoral artery and the superior mesenteric artery, leading to acute limb ischemia and acute mesenteric ischemia, respectively [10]. This patient underwent a femoral embolectomy that failed to salvage the limb, and he ultimately required an above-knee amputation. He also underwent a laparotomy with small bowel resection.

In contrast to the above cases, our patient had a successful outcome and survived to discharge with his limb preserved. This was due to early recognition and immediate surgical intervention. Popliteal embolectomy restored the blood supply in a timely manner and fasciotomy prevented reperfusion injury and compartment syndrome. We were fortunate that the limb ischemia was unilateral, rather than bilateral, and the occlusion site was more distal that is popliteal artery rather than femoral or common iliac artery. There was also no concomitant bowel ischemia in our case.

Cases of lung cancer tumor embolism that cause limb ischemia severe enough to warrant fasciotomy are extremely rare. The only other such case was published by Schreffler *et al*. in 2012, when they reported a lung adenocarcinoma that had invaded into the left atrium and embolized to the left external artery and right popliteal artery causing bilateral limb ischemia, which was also associated with left frontal lobe cortical infarcts, bilateral renal infarcts, and splenic infarcts [11]. This patient underwent bilateral femoral embolectomy and bilateral fasciotomies resulting in successful revascularization and discharge. This case is also like our case because tumor embolization has occurred at multiple arterial sites indicating tumor showering. In our case, tumor embolization occurred at the left popliteal artery, right renal artery, and superior mesenteric artery.

As seen from the above cases, lung cancer invasion into the left atrium via the pulmonary vein is a well-described mechanism of tumor embolism [12]. Embolization can occur spontaneously or be triggered by surgical manipulation during tumor resection [13]. As a tumor invades the pulmonary vein; it can fragment resulting in embolization [14]. The tumor embolus follows the normal circulatory pathway, passing through the left atrium and ventricle before being pumped into the systemic circulation.

This explains why lung cancer and atrial myxoma are the most common tumors to embolize, given their proximity to the heart [15]. The tumor embolus is a large mass of tissue and typically lodges at bifurcation sites, where the vessel diameter suddenly decreases.

In this case, the diagnosis of tumor embolus was primarily clinical and radiological as the histology did not comment on tumor burden. Given the underlying lung malignancy and presence of thrombosis at three separate arterial sites (superior mesenteric artery, right renal artery, and the left popliteal artery) on CT scan, the embolus is likely to represent tumor showering. The macroscopic appearance of the embolectomy specimen was also more consistent with lung tissue, as opposed to a fibrin blood clot.

Acute tumor embolism is a physical process and should be differentiated from the hypercoagulability associated with malignancy. Cancer induces a thrombotic state by activating the coagulation cascade. It causes increased secretion of procoagulant molecules and inflammatory cytokines [16]. Other mechanisms include endothelial damage, altered protein metabolism, and hemodynamic compromise leading to stasis [8].

Factors predisposing to tumor embolism include the size of the tumor, rate of growth of tumor, number of tumor cells, malignant potential, and poor differentiation of tumor cells [13] In our case, the tumor was large, necrotic, and locally invasive and was, therefore, more likely to embolize.

Although we managed to salvage our patient’s limb, he underwent major surgery with a high morbidity. He was in the later stages of limb ischemia and required a fasciotomy to prevent compartment syndrome. We were also fortunate that embolization to the SMA did not cause acute mesenteric ischemia, which has a high mortality. Therefore, it may be more beneficial to focus efforts on prevention of acute tumor embolism through risk stratification and prophylactic antithrombotic therapy in high-risk cases.

Current American Society of Hematology guidelines recommend anticoagulation treatment to treat venous thromboembolism in cancer patients [17]. It is arguable that once a tumor physically invades the bloodstream, it behaves like a thrombus and is at high risk of fragmentation, clustering, and embolization. In light of such cases, it may be an option to give these patients preventative antithrombotic therapy. We hypothesize that it may prevent concomitant distal thrombosis of an artery and potentially avoid an amputation, bowel resection, or significant morbidity in some cases.

Generally, acute tumor embolus has a poor prognosis due to the advanced stage of malignancy. Often, it is regarded as a preterminal event and palliative treatment may be considered more appropriate. Surgical intervention does not change the overall prognosis for the patient, which is determined by the stage and type of cancer.

This patient required emergency surgical embolectomy to save his limb. Catheter-directed thrombolysis was not appropriate in this case because the mechanism of action of thrombolytic agents involves binding to fibrin to activate tissue plasminogen to form plasmin, which is a fibrinolytic enzyme that dissolves the fibrin blood clot. As the embolus in this case was due to tumor, rather than a fibrin clot, thrombolysis would have been ineffective.

This case shows that surgical embolectomy can be immediately limb saving and prevent major morbidity for patients with acute tumor embolism causing limb ischemia. It can prevent complications such as compartment syndrome, rhabdomyolysis, and acute kidney injury. Therefore, it should be considered in carefully selected patients as it can greatly improve the patient’s quality of life in the terminal stages of disease.

## Conclusions

Tumor embolism resulting in popliteal occlusion is a rare occurrence and can cause loss of limb and death. Lung cancer invading into the pulmonary veins is associated with embolization to the systemic circulation. The clinician needs to be cognizant to the possibility of tumor embolism in such cases, as limb salvage depends on early diagnosis and surgical intervention.

Further research is needed to explore the role of prophylactic antithrombotic therapy in preventing tumor embolization. It would also be useful to compare prognostic outcomes with and without surgical intervention in patients with acute tumor embolism.

## Data Availability

The data supporting this study is with the author and has been included within the manuscript.

## Abbreviations

ATE: Acute tumor embolism
SMA: Superior mesenteric artery
CT: Computed tomography
CTA: Computed tomography angiogram
TNM-tumor: Node and metastases (cancer staging system)
